# Supplementary material for: A Comprehensive Assessment of Ultraviolet-Radiation-Induced Mutations in Flammulina filiformis Using Whole-Genome Resequencing
Source: J Fungi (Basel). 2024 Mar 20;10(3):228. doi: 10.3390/jof10030228 (PMC10971301; doi:10.3390/jof10030228)
Supplement: Supplementary file 1 [file jof-10-00228-s001.zip › Supplementary Material S8/KEGG annotation/out/64381550635650.os/KO/out_map/map04113.html]

KEGG PATHWAY: Meiosis - yeast - Reference pathway


|  |  |
| --- | --- |
| **Meiosis - yeast - Reference pathway** |  |

[
Pathway menu
| Organism menu
| Pathway entry
| Show description
| User data mapping
]

During meiosis, a single round of DNA replication is followed by two rounds of chromosome segregation, called meiosis I and meiosis II. At meiosis I, one pair of sister chromatids recombines with, then segregates from, its homologous pair, while the sister chromatids segregate from each other at meoisis II. In yeast, the switch to enter growth phase or meiosis phase is controlled by nutritional signals. At the onset of meiosis, nutritional signaling pathways converge on transcriptional regulation of Ime1. Ime1 is a transcriptional activator to promote expression of early meiosis-specific genes required for pre-meiotic DNA replication and meiotic recombination. Two of the early meiosis-specific genes, a transcriptional activator, Ndt80, and a CDK2 homolog, Ime2, are required for the transcription of middle meiosis-specific genes, which includes B-type cyclin genes and genes involved in spore wall formation. Polo-like kinase Cdc5, whose expression is activated by Ndt80, is required for the critical step in meiotic progression. Cdc5 regulates sister-kinetochore co-orientation by promoting the localization of Mam1 and Lrs4, components of the co-orientation complex, to kinetochores. Cdc5 also phosphorylates the meiotic cohesin subunit Rec8, which is essential for its cleavage and removal by separase. Moreover, Cdc5 promotes the release of protein phosphatase Cdc14 from the nuclezus, which is required for anaphase I spindle disassembly and exit from meiosis I. The centromeric protein Sgo1 is a key regulator of meiotic chromosome segregation. Sgo1 with PP2A may protect pericentromeric but not arm cohesion of sister chromatids during meiosis I. Sgo1 then disappears at meiosis II to allow sister chromatids to separate.

|  |  |
| --- | --- |
| Reference pathway | 100% |
